# Inclusion and Human Rights in Health Policies: Comparative and Benchmarking Analysis of 51 Policies from Malawi, Sudan, South Africa and Namibia

**DOI:** 10.1371/journal.pone.0035864

**Published:** 2012-05-23

**Authors:** Malcolm MacLachlan, Mutamad Amin, Hasheem Mannan, Shahla El Tayeb, Nafisa Bedri, Leslie Swartz, Alister Munthali, Gert Van Rooy, Joanne McVeigh

**Affiliations:** 1 Centre for Global Health, School of Psychology, Trinity College Dublin, Dublin, Ireland; 2 Centre for Rehabilitation Studies, Stellenbosch University, Stellenbosch, South Africa; 3 Research Administration, Ahfad University for Women, Omdurman, Sudan; 4 School of Psychology, Ahfad University for Women, Omdurman, Sudan; 5 School of Health Sciences, Ahfad University for Women, Omdurman, Sudan; 6 School of Psychology, University of Stellenbosch, Stellenbosch, South Africa; 7 Centre for Social Research, University of Malawi, Zomba, Malawi; 8 Multidisciplinary Research Centre, University of Namibia, Windhoek, Namibia; Johns Hopkins Bloomberg School of Public Health, United States of America

## Abstract

While many health services strive to be equitable, accessible and inclusive, peoples’ right to health often goes unrealized, particularly among vulnerable groups. The extent to which health policies explicitly seek to achieve such goals sets the policy context in which services are delivered and evaluated. An analytical framework was developed – *EquiFrame* – to evaluate 1) the extent to which 21 Core Concepts of human rights were addressed in policy documents, and 2) coverage of 12 Vulnerable Groups who might benefit from such policies. Using this framework, analysis of 51 policies across Malawi, Namibia, South Africa and Sudan, confirmed the relevance of all Core Concepts and Vulnerable Groups. Further, our analysis highlighted some very strong policies, serious shortcomings in others as well as country-specific patterns. If social inclusion and human rights do not underpin policy formation, it is unlikely they will be inculcated in service delivery. *EquiFrame* facilitates policy analysis and benchmarking, and provides a means for evaluating policy revision and development.

## Introduction

The global health movement has promoted the concept of health as a human right, globally, with the Alma Ata Declaration of ‘Health for All’ in 1978 being followed by calls for greater equity, accessibility and social inclusion over the last three decades [1]. The promotion and protection of health and human rights are inextricably linked: human rights violations may have severe health consequences; health policies and programmes may either protect or violate human rights in their design or implementation, such as the right to privacy; and vulnerability to ill-health may be decreased by acting to protect human rights, such as freedom from discrimination on grounds of ethnicity [2]. Public health is most effectively protected through the promotion of human rights and the protection of the inherent dignity of the person [3]. In recent years, international human rights laws have encouraged policy objectives to prioritize the health of the disadvantaged, so that health systems are effectively reorientated toward equity in healthcare. To promote ‘Health for All’, we therefore need to focus on *equitable healthcare* – that is, healthcare appropriate to peoples’ health needs, their personal situation and their broader socioeconomic context – rather than *equal healthcare* – where everybody gets the same [4]. Even with limited resources, services should aim for equity, emphasizing the individual and their dignity rather than their merits, economic circumstances or ethnicity [5].

 The extensive gap in access to healthcare between disparate groups in low as well as high-income countries is well established [6]. Non-discrimination implies that States must recognize and provide for the specific needs of groups that confront particular challenges through disaggregation of their health policies [7], [8]. Thus, to ensure equal opportunities for accessing health, health policies need to make particular efforts to address those who are less well positioned - physically, socially, culturally or economically - in and by society.

Selected factors to categorize groups should reflect specific subgroups of the population, such as poor rural women, or members of an ethnic minority, that require particular awareness due to their underlying social characteristics, which afford them less opportunity to be healthy than their more privileged counterparts [9]. Vulnerable groups may be defined as “social groups who experience limited resources and consequent high relative risk for morbidity and premature mortality” [10] and this may include children, the aged, ethnic minorities, displaced people, people suffering from some illnesses and persons with disabilities. Importantly, Eichler and Burke [11] have recognized that the social discrimination and bias that arises based on such categories is the result of social hierarchies: similar exclusionary practices disadvantage and disempower different groups, undermining their human rights and their rights to health, other social services and to social inclusion – to being full participants in society.

Progress towards the health-related Millennium Development Goals (MDGs) has, arguably, been achieved through being able to assist those who have had easier access to healthcare. Subsequent gains will be dependent on addressing the challenges faced by a range of vulnerable groups. The United Nations has been formative in highlighting the rights of various vulnerable or marginalized groups, including, for instance, the rights of displaced populations [12], children’s rights [13], and most recently the rights of persons with disabilities [14]. It is therefore important to establish to what extent these and related attempts to address social inequity, injustice and exclusion, over at least the past 30 years, have impacted on existing policies, and to develop a framework that can facilitate in policy analysis and, where necessary, policy formation and revision.

Perspectives on policy analysis differ. Stage Models examine the development of policy through stages or phases [15]. Network Frameworks examine interactions and interconnections between actors in the policy process [16]. Policy Space Analysis considers the broader policy context, circumstances and exigencies within which policy elites operate [17]. The Policy Triangle Framework [18] incorporates some aspects of the aforementioned approaches by considering the relationship between policy actors, content, process and context. While these approaches focus on the critical importance of how policy is made, they offer little guidance on evaluating policy ‘on the books’, that is, how, once formulated, policy should then be evaluated. Exworthy [15] emphasizes that while existing policy frameworks support the process of policy development, they do not provide a comprehensive appraisal of existing policies. Further, Gilson et al. [19] contend that policy analysis in low and middle-income countries is in urgent need of development and that many existing frameworks derive from high-income countries and are not necessarily easily applicable to other settings. They also call for the development of new methodologies and the use of comparative studies across countries.

This paper addresses each of these concerns by reporting on the development and application of a new standardized framework, *EquiFrame*. The principle aim is to demonstrate the use of a novel and reproducible methodology for using human rights as a framework for policy analysis. *EquiFrame* evaluates the degree of commitment of an existing policy to 21 Core Concepts of human rights and to 12 Vulnerable Groups. We sought to develop a framework that evaluates how coherent a policy is by developing “core concepts (that) informs the analyst concerning what the policy is, what it is intended to accomplish, and perhaps even what it does accomplish” [20], and to ascertain the extent of coverage of vulnerable groups in such policies. For instance, it has been argued that while the number of persons with disabilities is increasing globally, this is not reflected by the coverage of this group in relevant policies [21]. Accordingly, a particular interest of the research team was to assess the degree to which persons with disabilities (identified by *EquiFrame* as a Vulnerable Group) were incorporated in policy documents for the purpose of promoting more accessible healthcare.

We believe it is important to establish whether health policies include not only commitments to core concepts of human rights ‘for all’, but also whether these are promoted for vulnerable groups in a way which takes account of their ‘vulnerabilities’. In other words, it is important to know if human rights are promoted in health policies, and if so, if they are promoted in a socially inclusive way. *EquiFrame* allows the analyst to identify the strengths and weaknesses in current policy according to how stongly or weakly the policy advances core concepts of human rights in healthcare particularly among vulnerable groups. We sought to assess the extent to which health policy documents in four African countries with distinctive health challenges - Sudan, Namibia, Malawi, and South Africa - promoted equitable, accessible, and inclusive health services. Our goal was to identify, at the policy level, the extent to which existing health policies address the health-related human rights of vulnerable groups, distinguish best-practice policies and identify policies that are in need of urgent revision.

## Methods

### Development of *EquiFrame*

The World Health Report, ‘Working together for health’ [22], noted that Africa has the greatest disease burden of any continent but has the poorest health services. The four African countries that are the focus of this policy analysis framework each represent distinct challenges in terms of equitable access to healthcare. These four countries allow us to address how access to the healthcare systems for vulnerable groups can best be promoted in contexts where a large proportion of the population has been displaced (Sudan); where the population is highly dispersed (Namibia); where chronic poverty and high disease burden compete for meagre resources (Malawi); and where, despite relative wealth, universal and equitable access to healthcare is yet to be attained (South Africa).

With the intention of developing a health policy analysis framework that would be of particular relevance in low-income countries in general, and in Africa in particular, team members across Sudan, Malawi, Namibia, South Africa, Norway and Ireland, incorporating universities, research organizations and non-governmental organizations, undertook literature searches and discussions with relevant colleagues to identify potential frameworks that could address the principles of *universal*, *equitable* and *accessible* health services. Although we were not able to identify an ideal existing instrument, we drew on several existing approaches in the area. These included the core concepts of disability policy as developed by Turnbull and colleagues [20], [23]; the right to the highest attainable standard of health - and in particular the need to address health inequalities [24], [25] - and current thinking in health policy analysis more broadly [19], [26]. The Stowe and Turnbull approach, while specific to persons with disabilities and developed for use in North America, had many features relevant to our own interests. We, therefore, used some of the concepts they had identified, revised others and developed more from elsewhere. As indicated in the following section, the literature from which all of our core concepts of human rights were derived is identified in Table S1, and the basis for concept amalgamation is outlined.

Initial ideas for the framework were shared at a project meeting in Khartoum, and developed into a draft framework. The Draft Framework was presented at consultation workshops conducted in Sudan, Malawi, Namibia and South Africa and attended by over one hundred participants drawn from relevant clinicians and practitioners, civil servants, elected government representatives, non-governmental organizations (NGOs), independent consultants, researchers and academics, including members of different vulnerable groups. Feedback was incorporated into a revised Framework, following further discussion and removal of some overlapping terms and categories.

The Framework was then used to assess over 70 health policies drawn from the four African country partners, as well as African regional and international documents. The results from this analysis were then presented at Feedback Workshops in Sudan, Malawi, Namibia and South Africa, and the information gained from these workshops was incorporated into the Framework outlined below. The Framework presented here also benefited, significantly, from a workshop conducted for the Ministry of Health in Malawi, for the purpose of revising the Malawian National Health Policy [27], where novice users of the Framework gave feedback suggesting, for instance, simpler labels for Core Concepts and simpler definitions of those Concepts, to enhance user-friendliness. Finally, feedback from conference presentations and high level meetings has helped in shaping *EquiFrame* (for example, see 28–30].

*EquiFrame* has been developed as part of a Work Package led by Ahfad University for Women, Sudan, within a larger EU FP7 funded project, EquitAble, which is led by the Centre for Global Health at Trinity College Dublin, with a consortium of international partners (see www.equitableproject.org). Advisory groups to project EquitAble include Disability Studies scholars, who have reviewed the mapping of the Core Concepts and Vulnerable Groups incorporated in *EquiFrame* as well as the finalized version of the Framework. Feedback and expert advice, beyond our own project team (see www.equitableproject.org), from a variety of sources has, therefore, helped to shape and add authority and representativeness to the version of *EquiFrame* presented below.

### The Framework

*EquiFrame* evaluates the degree of stated commitment of an existing policy to 21 Core Concepts of human rights and to 12 Vulnerable Groups, guided by the ethos of universal, equitable and accessible health service provision. The Framework has been devised with the aim of generating a systematic evaluative and comparative analysis of health policies on technical content and design. *EquiFrame* allows the analyst to identify the strengths and weaknesses in current policy according to how strongly, or weakly, the policy advances the core concepts of human rights for health among vulnerable groups.

Our policy analysis framework was developed to ensure that researchers across our four countries explored different health policies from a common starting point, proceeding systematically and using a standard scoring system. The emergent *EquiFrame* methodology was used to analyze health policy documents in terms of coverage of Core Concepts and Vulnerable Groups included in the policy documents. Accordingly the framework (a) defines Core Concepts, (b) identifies the key questions and key language on which the Concept is based, (c) identifies Vulnerable Groups included, and (d) provides a data extraction matrix to chart the analyzed documents.

### Core Concepts

Core Concepts for relevant principles (*universal*, *equitable* and *accessible*) were identified and the available definitions were extracted from the above and related literature, resulting in 37 Core Concepts. Through group discussion, e-mail consultation with the Project Team, and stakeholder meetings, these concepts were refined and, where possible, integrated, resulting in 21 Core Concepts. These stakeholder meetings, held between April and July of 2009, were conducted in Sudan, Namibia, Malawi, and South Africa, and were established to deliberate the process and rationale for the inclusion of each Core Concept in *EquiFrame*. They were comprised of policy analysts and researchers from relevant ministries, including health and social affairs, and civil society organizations, including organizations of persons with disabilities. This reduction from 37 to 21 Core Concepts was necessary to make subsequent policy analysis manageable and to outline categories that were sufficiently discrete. Specifically, the Core Concept of ***Access***, utilised in the current framework, was derived from the consolidation of 8 preliminary Core Concepts corresponding to accessibility derived from the literature [31], [32], [33]; the Core Concept of ***Non-discrimination*** was derived from the synthesis of a further 6 concepts [20], [31], [32]; ***Capacity building*** was derived from the merging of 2 concepts [20], [33]; ***Cultural responsiveness*** was derived from the consolidation of 2 concepts [20], [31]; ***Protection from harm*** was derived from the synthesis of 2 concepts [32], [34]; and ***Individualized services*** was derived from the amalgamation of a further 2 concepts [32], [35]. The resulting 21 Core Concepts, grounded in international legal human rights instruments (see emboldened references of Table S1), were not established as necessarily being of equal importance but rather as representing a range of salient concerns to be addressed in striving for equitable, accessible and universal healthcare.

The Core Concepts were identified in existing health policies by two researchers who independently analyzed the documents. When a reference to a Core Concept was identified, the extent to which the Core Concept was addressed was ascertained using a series of key questions and key language (Table S1), each series tailored to elucidate the specified Core Concept.

### Vulnerable Groups

While the term ‘vulnerability’ is one of the most frequently used terms in social science research, difficulties arise when it comes to applying this concept as a tool for measurement and analysis. Vulnerable groups may be defined as social groups who experience limited resources and consequent high relative risk for morbidity and premature mortality [10], and rights approaches that prioritize those who are most vulnerable inherently promote equity by privileging those who are marginalized [36]. This definition of vulnerable groups chimes with the idea that vulnerability should be related to claims for special protection (for instance, in health policies), where there is a) a greater likelihood of people experiencing “wrongs”, and b) a duty to avoid identifiable “wrongs” [37].

The inclusion of vulnerable groups is an ethical imperative for health policy, requiring the development of appropriate indicators [38]. Furthermore, the social determinants approach to public health sees the identification of vulnerable population groups and the causes of differential vulnerability as being of critical importance, allowing us to sensitize vulnerable populations to the health benefits of programmes, extend service coverage and reduce barriers to access – all key components of inclusive health [39], [40]. However, quantifying vulnerability is challenging as is identifying just who is to be considered ‘vulnerable’. This concept needed to be clarified in order to reinforce its heuristic capacity, and political and practical relevance. To draw up a comprehensive list of appropriate social groups, we conducted a literature review spanning the international and national literatures. The resulting list was then refined and integrated to produce a categorization that would be credible across the four project countries, as well as regional and international health policies. However, it was evident that there was also a need for flexibility for the purpose of accommodating any additional country-specific groups, where integration of them into another theme might miss the opportunity to provide valuable information. Vulnerable Groups outlined by *EquiFrame* are provided in Table S2, and these resonate with the “Social Determinants Approaches to Public Health” report [39].

### Scoring

A data extraction matrix (checklist) was developed to measure the quality of the analyzed policy documents. The *EquiFrame* Matrix was constructed with the vertical axis listing the 21 Core Concepts and the horizontal axis listing the 12 or more Vulnerable Groups.

Each Core Concept received a score on a continuum from 1 to 4. This was a rating of the quality of commitment to the Core Concept within the policy document:

1 = Concept only mentioned.

2 = Concept mentioned and explained.

3 = Specific policy actions identified to address Concept.

4 = Intention to monitor Concept was expressed.

If a Core Concept was not relevant to the document context, it was stated as not applicable.

Each policy document was assessed by two independent raters. Inter-rater reliability was established through the comparison of evaluations by raters subsequent to separately analyzing a relevant policy document. In each document the presence of Core Concepts was assessed for each Vulnerable Group that was identified in the policy. If no Vulnerable Group was mentioned but a Core Concept addressed the total population (e.g. “all people”), the Core Concept was scored as ‘Universal’. The total number and scores for mentioned Core Concepts and Vulnerable Groups was calculated for each document across the four countries. Where differences of interpretation occurred these were addressed by subsequent discussion until a consensus position was agreed between raters.

### The 4 Summary Indices of *EquiFrame* are Outlined Below:

1. Core Concept Coverage. A policy was examined with respect to the number of Core Concepts mentioned out of the 21 Core Concepts identified; and this ratio was expressed as a rounded up percentage. In addition, the actual terminologies used to explain the Core Concepts within each document were extracted to allow for future qualitative analysis and cross-checking between raters [41]–[44].
2. Vulnerable Group Coverage. A policy was examined with respect to the number of Vulnerable Groups mentioned out of the 12 Vulnerable Groups identified: and this ratio was expressed as a rounded up percentage. In addition, the actual terminologies used to describe the Vulnerable Groups were extracted to allow for qualitative analysis and cross-checking between raters [41]–[44].
3. Core Concept Quality. A policy was examined with respect to the number of Core Concepts within it that were rated as 3 or 4 out of the 21 Core Concepts identified; that is, as either stating a specific policy action or intention to monitor that action. When several references to a Core Concept were found to be present, the top quality score received was recorded as the final quality scoring for the respective Concept.
4. Overall Summary Ranking.Each document was given an Overall Summary Ranking in terms of it being of High, Moderate, or Low standing according to the following criteria:
  - High = if the policy achieved ≥50% on all of the three scores above.
  - Moderate = if the policy achieved ≥50% on two of the three scores above.
  - Low = if the policy achieved <50% on two or three of the three scores above.

### Selection of Policies

Health ‘policies’ were defined as ‘courses of action (and inaction) that affect the set of institutions, organizations, services and funding arrangements of the health system’ [45]. Health policies were included if they met the following criteria:

1. Health policy documents produced by the Ministry of Health
2. Policies addressing health issues outside of the Ministry of Health
3. Strategies that address health policies
4. Policies related to the top 10 health conditions identified by WHO

#### [Malawi

HIV/AIDS; Lower respiratory infections; Malaria; Diarrhoeal diseases; Perinatal conditions; cerebrovascular disease; Ischaemic heart disease; Tuberculosis; Road traffic accidents; Protein energy malnutrition.

#### Namibia

HIV/AIDS; Perinatal Conditions; Cerebrovascualar disease; Tuberculosis; Ishaemic heart disease; Diarrhoeal disease; Malaria; Violence; Lower respiratory infections; Road traffic accidents.

#### South Africa

HIV/AIDS; Cerebrovascular disease; Ischaemic heart disease: Violence; Tuberculosis; Diarrhoeal diseases; Road traffic accidents; Diabetes mellitus; Chronic obstructive pulmonary disease.

#### Sudan

Schaemic heart disease; Malaria; HIV/AIDS; Diarrhoeal diseases; Measles; Tuberculosis; Cerebrovascular disease; Perinatal conditions; War; Road traffic accidents.]

A search was carried out to locate available health policies. The relevant ministries, agencies, and libraries were contacted and asked to identify policy documents falling within the scope of our research. The policy documents meeting the inclusion criteria in the four countries were: Malawi, 14; Namibia, 10; South Africa, 11; and Sudan, 16. We sought to assess the extent to which health policy documents in Sudan, Namibia, Malawi and South Africa promoted equitable, accessible and inclusive health services.

## Results

**Figure 1:**
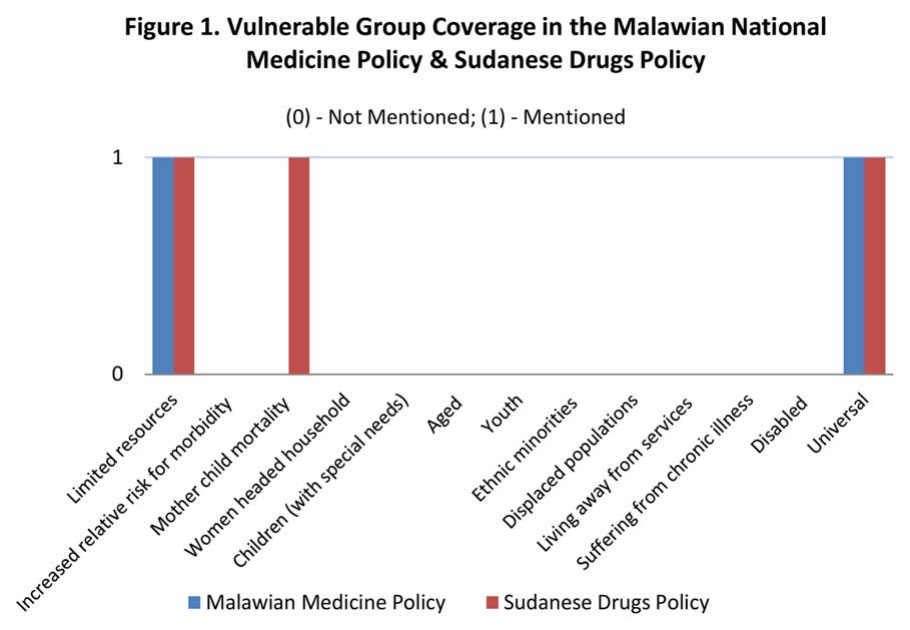
Vulnerable Group Coverage in the Malawian National Medicine Policy and Sudanese Drugs Policy. [Both the Malawian National Medicine Policy and the Sudanese Drugs Policy were assessed by two researchers who independently analyzed the documents in terms of Vulnerable Group Coverage. These policies were assessed with respect to the number of Vulnerable Groups mentioned out of the 12 Vulnerable Groups identified. If no Vulnerable Group was mentioned but a Core Concept addressed the total population (e.g. “all people”), the Core Concept was scored as ‘Universal’. This ‘Universal’ scoring was not however included in the calculation for overall Vulnerable Group Coverage.].

**Figure 2:**
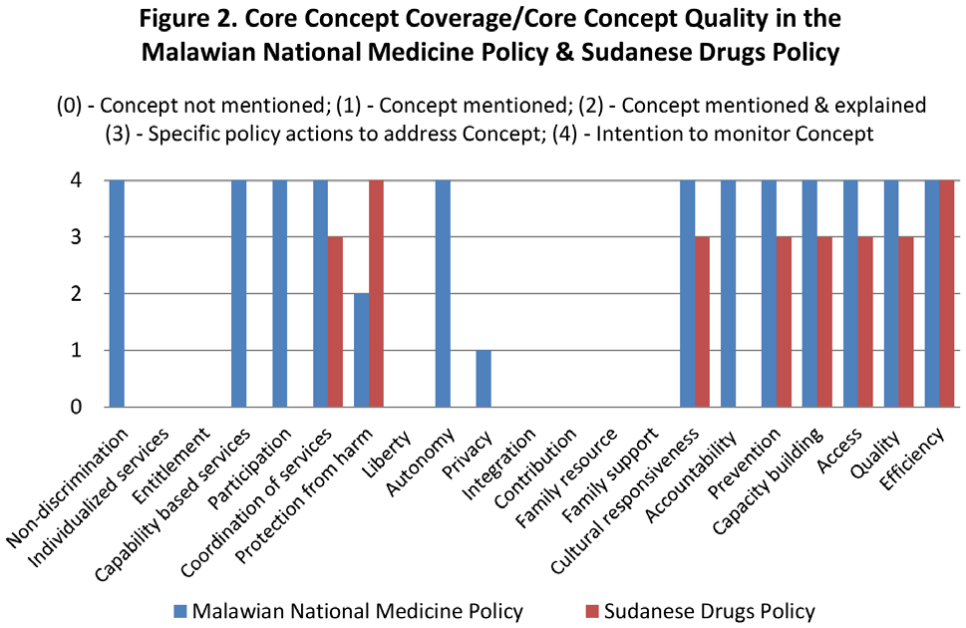
Core Concept Coverage/Core Concept Quality in the Malawian National Medicine Policy and Sudanese Drugs Policy. [The Malawian National Medicine Policy and the Sudanese Drugs Policy were assessed by two researchers who independently analyzed the documents in terms of Core Concept Coverage and Core Concept Quality. These policies were assessed with respect to the number of Core Concepts mentioned out of the 21 Core Concepts identified (Core Concept Coverage). When a reference to a Core Concept was identified, the extent to which the Core Concept was addressed was ascertained using a series of key questions and key language, each series tailored to elucidate the specified Core Concept. Each Core Concept also received a score on a continuum from 1 to 4 (Core Concept Quality). This was a rating of the quality of commitment to the Core Concept within the policy document: (1) Concept only mentioned; (2) Concept mentioned and explained; (3) Specific policy actions identified to address Concept; (4) Intention to monitor Concept expressed.].

To illustrate more detailed output from *EquiFrame*, Figures 1 and 2 illustrate *EquiFrame* as applied to just two of the policies that were analyzed; the Malawian National Medicine Policy and Sudanese Drugs Policy. Each of these polices performed poorly on Vulnerable Group Coverage. Only those with *Limited Resources* were mentioned in both, and *Mother Child Mortality* was mentioned only in the case of Sudan. None of the remaining Vulnerable Groups were mentioned, while ‘universal’ terms, such as “all people” were used more than 200 times in the Malawian policy and just above 20 times in the Sudanese policy. Core Concept Coverage and Core Concept Quality varied more dramatically between the two countries. For instance, in the Malawian policy, *Autonomy*, *Participation* and *Non-discrimination* were mentioned and an intention to monitor was expressed. These Concepts were not mentioned in the Sudanese policy however. Both Core Concept Coverage and Core Concept Quality were greater in the Malawian policy (66% and 57% respectively) than in the Sudanese policy (38% and 38% respectively). As both policies mentioned far fewer than 50% of the possible Vulnerable Groups, neither scored *High* on our Overall Summary Ranking index; the Malawian policy scored *Moderate* (having exceeded 50% on two indices), while the Sudanese policy scored *Low* (failing to exceed 50% on all three indices). This example should assist the reader in the interpretation of the main results summarized in Tables 1, 2, 3, 4.

**Table 1:** The overall quality assessment of policies analyzed: Malawi.

Policies	VG%	CC%	% of CC qualitybetween 3 to 4	Quality of the policy
National HIV Policy	66.7	81.0	42.8	Moderate
National Medicine Policy	8.3	66.7	57.1	Moderate
National Policy On Equalisation Of Opportunities For Persons With Disabilities	16.7	57.1	42.8	Low
National Mental Health Policy	33.3	66.7	47.6	Low
Malawi Policy On Tuberculosis Control In Prisons	25	52.4	38	Low
Traditional Medicine Policy	8.3	61.9	42.8	Low
Injection Safety Policy	16.7	61.9	42.8	Low
National Health Policy	8.3	71.4	19.0	Low
Policy On Equity In Access To Antiretroviral Therapy (Art) In Malawi	16.7	42.9	9.5	Low
National Sexual And Reproductive Health And Rights (SRHR) Policy	25	71.4	23.8	Low
Malaria Policy	25	52.4	38	Low
National Policy On Orphans And Other Vulnerable Children	8.3	61.9	33.3	Low
IMCI Approach Policy For Accelerated Child Survival And Development In Malawi	8.3	52.4	23.8	Low
Infection Prevention And Control Policy	0	47.6	14.3	Low

**Table 2:** The overall quality assessment of policies analyzed: Namibia.

Policies	VG%	CC%	% of CC qualitybetween 3 to 4	Quality of the policy
National Reproductive and child health policy	83	90	62	High
National Gender Policy	75	71	52	High
National Policy for Mental health	58	71	57	High
National Policy on Disability	58	95	57	High
National guidelines for the management of Tuberculosis	33	80	76	Moderate
National Policy on HIV/AIDS	75	100	43	Moderate
Policy on Orthopaedic Technical Services	50	66	48	Moderate
National Malaria Policy	25	43	28	Low
Control of Acute Respiratory Infections (ARI) Programme	25	43	10	Low
Integrated Management of Childhood Illness (Diarrhoea)	25	24	24	Low

**Table 3:** The overall quality assessment of policies analyzed: South Africa.

Policies	VG%	CC%	% of CC qualitybetween 3 to 4	Quality of the policy
The HIV and AIDS and STI Strategic Plan for SA 2007–2011	66.6	80.95	66.66	High
The White Paper for the Transformation of the Health System	50	52.38	42.86	Moderate
Tuberculosis Strategic Plan for SA 2007–2011	50	62	47.62	Moderate
Strategic Plan 2009/10–2011/12	41.6	57.14	38.09	Low
Strategic Priorities for the National Health System 2004–2009	41.6	42.86	9.52	Low
Policy on Quality in Health Care for SA	33.3	14.29	0	Low
The National Rehabilitation Policy	41.6	47.62	19.04	Low
The National Programme for control and management of DiabetesType 2 at primary level	25	38.09	4.76	Low
The South African Hypertension Guideline 2006	33.3	19.05	4.76	Low
The National Guide on Stroke and Transient Ischaemic Attack Management	25	14.29	9.52	Low
The Guidelines for Cholera Control	25	23.81	4.76	Low

**Table 4:** The overall quality assessment of policies analyzed: Sudan.

Policies	VG%	CC%	% of CC qualitybetween 3 to 4	Quality of the policy
National Health policy	83	67	52	High
South Sudan Health policy	75	62	57	High
Mental Health policy	92	86	48	Moderate
Disaster policy	75	57	29	Moderate
Health promotion strategy	75	52	24	Moderate
Non-Communicable diseases	92	62	38	Moderate
Nutrition policy	67	57	29	Moderate
Reproduction health policy	50	71	29	Moderate
Women empowerment policy	17	29	10	Low
Voluntary sector policy	0	29	5	Low
TB policy	42	57	29	Low
Malaria policy	42	38	29	Low
AIDS policy	25	71	33	Low
Private sector policy	0	52	19	Low
Drugs policy	17	38	38	Low
Disability policy	42	62	24	Low

Having illustrated more detailed output from a selection of two policies analyzed, namely the Malawian National Medicine Policy and Sudanese Drugs Policy, the application of *EquiFrame* to all 51 policies analyzed across Malawi, Namibia, South Africa and Sudan will now be briefly discussed. All Core Concepts were mentioned in at least one of the policies analyzed across the four countries. This lends support to the construct validity of the categories used, as they appear to have relevance within the policy domain, at least across the policies studied here. The most frequently mentioned Vulnerable Groups were considered across comparable policies in the four countries. These comparable policies were on HIV/AIDS, Tuberculosis and Disability. Those Vulnerable Groups most mentioned across these common policies were *Disabled* persons, persons *Suffering from Chronic Illness* and *Youth*. Indeed the prominence of *Disabled* persons and persons *Suffering from Chronic Illness* within the Disability and TB policies respectively is to be expected, and thus supports the internal validity of the *EquiFrame* methodology, at least as applied to these policies. However, is notable that the HIV/AIDS and TB policies most frequently mentioned what we termed a ‘universal’ group; for instance, the policy would refer to “all people”, or “everyone”. While reference was made to a universal grouping in the Malawian and Sudanese Disability policies (although it was not the most frequent), no mention at all of any universal group was made in the South African or Namibian Disability policies. These results are indicative of the variation both between countries and policies, but also signify the prominence of certain Vulnerable Groups.

Having indicated that the framework can be used reliably between different raters and that it presents aspects of construct and internal validity, we now consider how national policies performed relative to others in terms of the summary indices described above. These results are presented in Tables 1, 2, 3, 4. With the exception of Malawi which failed to have any policies that rated as *High* quality, all countries had policies rated in each of the *High*, *Moderate* and *Low* ranges and each country differed in the proportion of policies falling in each of these ranges. The results for each individual country are now briefly discussed.

### Malawi

Of the fourteen Malawian analyzed, none were assessed to be of *High* quality. Two were scored as *Moderate,* and twelve were scored as *Low* (see Table 1). Both the National HIV policy and National Medicine Policy were scored as *Moderate* quality. It is noteworthy that over 65% of Vulnerable Groups were mentioned in the National HIV policy, as no other Malawian policy exceeded the required criterion of 50% for this rating. Across all of the Malawian policies, Core Concept Coverage exceeded Vulnerable Group Coverage, with the lowest coverage of Core Concepts being slightly above 40% for the Policy on Equity in Access to Antiretroviral Therapy (Art) and the lowest coverage of Vulnerable Groups being 0% for the Infection Prevention and Control policy. With respect to Core Concept Coverage, twelve of the fourteen policies analyzed exceeded the required criterion of 50% for this rating. Quality of commitment to Core Concepts varied considerably however: Core Concept Quality was 57% for the National Medicine Policy, the only Malawian policy to exceed the required criterion of 50% for this rating, while this score was less than 10% for the Policy on Equity in Access to Antiretroviral Therapy (Art) policy.

### Namibia

Three of the ten Namibian policies analyzed were assessed as *Low* quality, while three were scored as *Moderate* quality. Four policies analyzed achieved an overall *High* rating (Table 2). These were the policies pertaining to Reproductive and Child Health, Gender, Mental Health and Disability. On these and several other policies over 70% of Core Concepts were included, and in the case of the HIV/AIDS policy 100% of Core Concepts were addressed. Core Concept Coverage again exceeded Vulnerable Group Coverage in all polices with the exception of the National Gender policy and the policy on the Integrated Management of Childhood Illness, which scored very poorly across all of our matrices. Core Concept Quality varied quite significantly in Namibia, from only 10% for Control of Acute Respiratory Infections (Ari) Programme up to 76% for Tuberculosis policy.

### South Africa

Only one South African document of the eleven documents analyzed – the HIV and AIDS and STI Strategic Plan – scored *High* overall according to our criteria (Table 3). Two policies was scored as *Moderate* while a further eight policies were scored as *Low* quality. Several documents had quite low scores for Vulnerable Group Coverage and Core Concept Coverage. Both Vulnerable Group Coverage and Core Concept Coverage were however highly variable across policies; from 25% to 66% for coverage of vulnerable groups and from 14% to 80% for coverage of core concepts. Core Concept Quality was very low across a number of documents. This included the National Programme for Control and Management of Diabetes Type 2 at Primary Level and the South African Hypertension Guideline, each scoring just below 5%, while the Policy on Quality in Health Care actually scored 0%, the only document to do so for this summary index across the 51 documents analyzed.

### Sudan

In total, sixteen Sudanese policy documents were analyzed. Both the National Health Policy (effectively the ‘Northern Sudan’ policy) and the South Sudan Health Policy scored in our *High* category. Six policies were scored as *Moderate*, while eight polices were scored as *Low* quality. Sudan presented the greatest range with regard to Vulnerable Group Coverage (Table 4). While 92% of vulnerable groups were mentioned in the Mental Health policy and Non-Communicable Diseases policy, 0% was mentioned in the Voluntary Sector policy or the Private Sector policy. Core Concept Coverage was also somewhat variable: in each of the three documents on Mental Health, on Reproductive Health, and on AIDS, Core Concept Coverage was over 70%, while this score fell to below 30% for the Women Empowerment policy and the Voluntary sector policy. With regards to Core Concept Quality, only the National Health Policy and South Sudan Health Policy exceeded our criterion of 50% for this rating. Particularly noteworthy was the strong performance of the Mental Health Policy in terms of Vulnerable Group Coverage (92%) and Core Concept Coverage (86%), although Core Concept Quality fell below our criterion (48%). By contrast, the Voluntary Sector Policy scored particularly poorly in terms of Vulnerable Group Coverage (0%), Core Concept Coverage (29%), and Core Concept Quality (5%).

## Discussion

Our analysis has highlighted some very strong health policies across Namibia, Malawi, Sudan and South Africa, serious shortcomings in others as well as country-specific patterns. The health sectors of each of these States face significant challenges in addressing inequities found to be present within a number of current African health policies. The foremost results of the study support existing literature that while the number of persons with disabilities is increasing globally, this is not reflected by the coverage of this group in relevant policies [21]. This paper has sought to present an overview of the framework and provide a comparative and benchmarking analysis. For further details specific to *EquiFrame*, and the process of its formulation, readers are referred to the *EquiFrame* manual [41, see also 42–44]. Both through the process of undertaking this research and feeding-back the results to stakeholder workshops in each of the four countries, we have noted several factors that are important to consider when interpreting results, either within or across countries. While the inclusion criteria sought the relevant policy documents in each country, not all of the documents analyzed were official ‘policies’; some were described as ‘guidelines’, or ‘strategic plans’, or ‘programmes’. Clearly these instruments may not have been designed with an equivalent purpose and so in some cases it may be misleading to deem them as being policy-related or to compare them, even in the absence of a policy document in that area. To the extent that such documents are not policy-related, one could simply highlight the lack of a policy.

The indices we have used – scores of over 50% for each of our ratings – could be altered to reflect different weighting or sensitivity with regard to human rights, vulnerability or specific actions to address a concept or intention to monitor a concept being expressed. Indeed these latter two categories could be treated separately rather than combined, as we did here. Ultimately *EquiFrame* is a methodology for descriptive analysis that can provide quantitative indices that can be fine-tuned for the required purpose.

Even when there may be strong comparability between the structure and function of policy instruments it may be that it is less reasonable to expect some documents to address human rights and vulnerable groups than others. For instance, is it reasonable for the Sudanese Voluntary Sector Policy (0%) and the Mental Health Policy (92%) to each mention vulnerable groups? It could be argued that one is about how a sector operates while the other is about provision of specific services. Even if one accepts this argument we feel that it can still be illuminating to know the extent to which they focus on social inclusion. In the case of Sudan, more comparable sector policies (National Health Policy, 83%) and service provision policies (Malaria Policy, 42%) also different considerably with regard to social inclusion.

A legitimate question is whether all vulnerable groups are equally salient across all types of policies. While this is certainly debatable, we feel that it is important to at the very least be able to make comparisons regarding the inclusion of vulnerable groups across different policy areas and then to consider the contextual relevance of these for the particular policies – without such data, we simply can’t make the comparisons. It may also be the case that certain assumptions lead to conceptual foreclosure when analyzing certain policies. For instance, it could be argued that HIV/AIDS policies necessarily address the *Increased relative risk for morbidity* group and thus it makes little sense to evaluate if such policies address this group. However, the high comorbidity of HIV/AIDS and TB particularly in sub-Saharan Africa [46]–[49] illustrates the value of doing so; policies that do not include groups with other serious co-morbid conditions would be less useful than those that do. Another and this time perhaps counter-intuitive example may be that a policy on the Integrated Management of Childhood Illness could not be expected to address Group 6 (*Aged*). However, a significant role of the elderly in the management of childhood illness is evident, in particular the role of mothers-in-law in decisions to seek treatment for sick children [50], the role of older women’s pensions in rural South Africa, where HIV/AIDS morbidity and mortality are having significant effects on household resources [51], and the pivotal role, as ‘Africa’s newest mothers’, that older people now play in the economic, social and psychological welfare of a proliferating number of orphaned and vulnerable children as a result of HIV/AIDS in Africa [52].

In our country feedback workshops, some stakeholders argued that some documents use the term “all”, as in “all people” to be fully inclusive and therefore reference to specific vulnerable groups is not necessary. Indeed, subsidiary analysis of the use of “all”, or its synonyms, indicates that documents using such ‘all-inclusive’ terms, also specify certain vulnerable groups, but not others. Accordingly, we feel it is important to establish which vulnerable groups are included, and which are not, as the use of inclusive terminology does not necessarily address the concerns of specific vulnerable groups.

While *EquiFrame* has been developed for the purposes of policy analysis, we do believe that its form of analysis can also be usefully applied to other types of planning and guiding documents, and that coverage of Core Concepts of human rights and inclusion of Vulnerable Groups is pertinent to a range of diverse guiding documents too. Fuller understanding of the content of any such documents can always be and should always be strengthened by understanding of the context in which the document was developed as well as the process of its development. However, describing ‘policy on the books’ is not only a legitimate practice, but a vital one, if we are to recognize and develop documents that are most likely to support human rights and promote greater inclusion in health service provision. It is also important to stress that while we have gone to considerable lengths in the consultation and development of *EquiFrame* to authenticate the Core Concepts and Vulnerable Groups described, we are not necessarily claiming that these are universally applicable. Rather that the process of deriving these concepts and vulnerable groups, is one that can be used in other settings and contexts to achieve similar ends.

Finally, while we have described the analysis of existing documents across Malawi, Namibia, South Africa and Sudan, it is hoped that the utility of *EquiFrame*, as a policy analysis tool, will extend beyond its application as a framework for evaluation to the development of new policy documents and to the revision of existing documents. By highlighting some high quality health policy documents, *EquiFrame* can navigate those developing policies towards some supreme examples of human rights coverage and vulnerable group inclusion. It can also provide a check-list of factors for consideration, as well as indicating specific terms and phrasing for use in a policy.

The extensive gap in access to healthcare between disparate groups in developing as well as developed countries is well established [6]. In the context of low-income countries, where resources are scarce, marginalised or vulnerable people may experience greater social exclusion with the result that their right to health is undermined to an even greater extent than in wealthier countries. Even with limited resources, services should aim for equity, emphasizing the individual and their dignity rather than their merits, economic circumstances or ethnicity [5]. Equity in healthcare is an astute and feasible political aspiration. If human rights and social inclusion do not underpin policy formation, it is unlikely they will be inculcated in service delivery however. Through its discernment of policy commitment to core concepts of human rights and vulnerable groups, guided by the principles of universal, equitable, and accessible health services, *EquiFrame* promises to promote the United Nations’ call for *Health for All,* with its implicit assumption of universal and equitable access to healthcare.

## Supporting Information

Table S1 ***EquiFrame*** **Key Questions and Key Language of Core Concepts.**
(DOCX)

Table S2 ***EquiFrame*** **Vulnerable Group Definitions.**
(DOCX)
