# Supplementary material for: Inclusion and Human Rights in Health Policies: Comparative and Benchmarking Analysis of 51 Policies from Malawi, Sudan, South Africa and Namibia
Source: PLoS One. 2012 May 23;7(5):e35864. doi: 10.1371/journal.pone.0035864 (PMC3359320; doi:10.1371/journal.pone.0035864)
Supplement: Table S1 — EquiFrame Key Questions and Key Language of Core Concepts. (DOCX) [file pone.0035864.s001.docx]

Table S1: *EquiFrame* Key Questions and Key Language of Core Concepts

| **Supporting Literature** | **Key Language** | **Key Question** | **Core Concept** | **No.** |
| --- | --- | --- | --- | --- |
| 1, 2, **3**, 4, 5, 6, **7**, **8**, **9**, **10**, **11**, **12** | Vulnerable groups are not discriminated against on the basis of their distinguishing characteristics (that is, Living away from services; Persons with disabilities; Ethnic minority or Aged). | Does the policy support the rights of vulnerable groups with equal opportunity in receiving health care? | **Non-discrimination** | 1. |
| **3**, 4, **7**, 13, **14**, **15**, 16, **17**, **18** | Vulnerable groups receive appropriate, effective, and understandable services. | Does the policy support the rights of vulnerable groups with individually tailored services to meet their needs and choices? | **Individualized Services** | 2. |
| **3**, 4, **8**, **14**, **15**, 19, 20, 21, **22**, **23** | People with limited resources are entitled to some services free of charge or persons with disabilities may be entitled to respite grant. | Does the policy indicate how vulnerable groups may qualify for specific benefits relevant to them? | **Entitlement** | 3. |
| **15**, **17**, 24, 25, **26**, **27**, **28** | For instance, peer to peer support among women headed households or shared cultural values among ethnic minorities. | Does the policy recognize the capabilities existing within vulnerable groups? | **Capability based Services** | 4. |
| **3**, 4, **10**, **11, 12,** 19, **29**, 30, 31, 32, 33, 34, 35, 36, 37, 38, 39, 40, 41, 42, **43**, **44**, **45** | Vulnerable groups can exercise choices and influence decisions affecting their life. Such consultation may include planning, development, implementation, and evaluation. | Does the policy support the right of vulnerable groups to participate in the decisions that affect their lives and enhance their empowerment? | **Participation** | 5. |
| **3**, 4, 6, **15**, 19, 20, 24, **29**, 41, 46, 47, 48, 49, **50**, **51**, **52** | Vulnerable groups know how services should interact where inter-agency, intra-agency, and inter-sectoral collaboration is required. | Does the policy support assistance of vulnerable groups in accessing services from within a single provider system (interagency) or more than one provider system (intra-agency) or more than one sector (inter-sectoral)? | **Coordination of Services** | 6. |
| **3**, **14**, **15**, **22**, **26**, **43**, **53** | Vulnerable groups are protected from harm during their interaction with health and related systems. | Are vulnerable groups protected from harm during their interaction with health and related systems? | **Protection from harm** | 7. |
| 4, **7**, **9**, **14**, **15**, **22**, 24, 30, 42, **54** | Vulnerable groups are protected from unwarranted physical or other confinement while in the custody of the service system/provider. | Does the policy support the right of vulnerable groups to be free from unwarranted physical or other confinement? | **Liberty** | 8. |
| **3**, 4, **9**, **10**, **29**, 30, **44**, **45**, **50**, **53**, 55, 56 | Vulnerable groups can express “independence” or “self-determination”. For instance, person with an intellectual disability will have recourse to an independent third party regarding issues of consent and choice. | Does the policy support the right of vulnerable groups to consent, refuse to consent, withdraw consent, or otherwise control or exercise choice or control over what happens to him or her? | **Autonomy** | 9. |
| 1, **3**, **9**, **15**, **22**, **50**, **54**, 57 | Information regarding vulnerable groups need not be shared among others. | Does the policy address the need for information regarding vulnerable groups to be kept private and confidential? | **Privacy** | 10. |
| 4, **15**, **22**, **23**, **27**, 41, **53**, **58** | Vulnerable groups are not barred from participation in services that are provided for general population. | Does the policy promote the use of mainstream services by vulnerable groups? | **Integration** | 11. |
| **15**, **22**, **26**, 41, **43**, **53**, 59, **60** | Vulnerable groups make a meaningful contribution to society. | Does the policy recognize that vulnerable groups can be productive contributors to society? | **Contribution** | 12. |
| **3**, 4, **8**, **14**, **15**, **17**, **23**, 24, 42, 49, **61** | The policy recognizes the value of family members of vulnerable groups as a resource for addressing health needs. | Does the policy recognize the value of the family members of vulnerable groups in addressing health needs? | **Family Resource** | 13. |
| 4, **7**, **9**, **14**, **15**, **26**, 41, 42, **53**, **62** | Persons with chronic illness may have mental health effects on other family members, such that these family members themselves require support. | Does the policy recognize individual members of vulnerable groups may have an impact on the family members requiring additional support from health services? | **Family Support** | 14. |
| 1, **3**, 4, 6, **10**, **15**, 19, 21, **22**, 34, 41, **44**, 48, 56, **60**, 63, 64 | i) Vulnerable groups are consulted on the acceptability of the service provided.  ii) Health facilities, goods and services must be respectful of ethical principles and culturally appropriate, that is, respectful of the culture of vulnerable groups. | Does the policy ensure that services respond to the beliefs, values, gender, interpersonal styles, attitudes, cultural, ethnic, or linguistic, aspects of the person? | **Cultural Responsiveness** | 15. |
| 1, **3**, 4, 6, **10**, **15**, **26**, 33, 42, **43**, 47, 48, **50**, 56, 57, 65, 66, 67 | Vulnerable groups have access to internal and independent professional evaluation or procedural safe guard. | Does the policy specify to whom, and for what, services providers are accountable? | **Accountability** | 16. |
| 1, **3**, **14**, **15**, **29**, 41, **50**, **53**, 68 |  | Does the policy support vulnerable groups in seeking primary, secondary, and tertiary prevention of health conditions? | **Prevention** | 17. |
| **3**, 4, **15**, **23**, 41, 42, 47, 48, **50**, **51**, 55, 69, **70** |  | Does the policy support the capacity-building of health workers and of the system that they work in addressing health needs of vulnerable groups? | **Capacity Building** | 18. |
| 1, **3**, 6, **8**, **10**, **14**, 19, 41, **43**, 48, **53**, **54**, 56, 57, 71, 72, 73, 74, 75, 76, 77, 78 | Vulnerable groups have accessible health facilities (that is, transportation; physical structure of the facilities; affordability and understandable information in appropriate format). | Does the policy support vulnerable groups –physical, economic, and information access to health services? | **Access** | 19. |
| 1, **3**, 6, **15**, 19, 21, 41, 56, 57, 64, 66, 75, 79, 80, 81, **82**, **83**, **84**, **85** | Vulnerable groups are assured of the quality of the clinically appropriate services. | Does the policy support quality services to vulnerable groups through highlighting the need for evidence-based and professionally skilled practice? | **Quality** | 20. |
| 6, **29**, **50**, **84**, 86, 87, **88**, **89**, **90** |  | Does the policy support efficiency by providing a structured way of matching health system resources with service demands in addressing health needs of vulnerable groups? | **Efficiency** | 21. |

**References**

1. Office of the United Nations High Commissioner for Human Rights, World Health Organization (2008) The Right to Health. United Nations, Geneva.
2. Braveman P (2006) Health disparities and health equity: Concepts and measurement. Annu Rev Public Health 27: 167-94.
3. United Nations Economic and Social Council (2000) Substantive Issues Arising in the Implementation of the International Covenant on Economic, Social and Cultural Rights. General Comment No. 14. The Right to the Highest Attainable Standard of Health (Article 12 of the International Covenant on Economic, Social and Cultural Rights). Available: <http://www.unhchr.ch/tbs/doc.nsf/%28symbol%29/E.C.12.2000.4.En>. Accessed: April 29, 2012.
4. Turnbull HR, Stowe MJ (2001) A taxonomy for organizing the core concepts according to their underlying principles. Journal of Disability Policy Studies 12(3): 177-97.
5. MacLachlan M (2006) Culture & health: A critical perspective towards global health (Second Edition). Chichester: Wiley.
6. Hunt P, Backman G (2008) Health systems and the right to the highest attainable standard of health*.* Health & Human Rights 10(1): 81-92.
7. African Union (1981) African (Banjul) Charter on Human and Peoples’ Rights. Available <http://www.africa-union.org/official_documents/treaties_%20conventions_%20protocols/banjul%20charter.pdf>. Accessed: March 20, 2012.
8. United Nations (1979) Convention on the Elimination of All Forms of Discrimination Against Women. Available: <http://www.un.org/womenwatch/daw/cedaw/cedaw24/cedawcegy45.pdf>. Accessed March 10, 2012.
9. United Nations (1966) International Covenant on Civil and Political Rights. Available: <http://treaties.un.org/doc/Publication/UNTS/Volume%20999/volume-999-I-14668-English.pdf>. Accessed: March 20, 2012.
10. United Nations (1993) Vienna Declaration and Programme of Action. Available: <http://daccess-dds-ny.un.org/doc/UNDOC/GEN/G93/142/33/PDF/G9314233.pdf?OpenElement>. Accessed: March 20, 2012.
11. United Nations (2000) United Nations Millennium Declaration. Available: <http://www.un.org/millennium/declaration/ares552e.htm>. Accessed March 20, 2012.
12. Social Services Commission, United States Government, Washington, D.C. (1990) Americans with Disabilities Act of 1990, 42 U.S. Code Chapter 126. Available: <http://www.ada.gov/pubs/adastatute08.pdf>. Accessed: March 21, 2012.
13. MacLachlan M, Mannan H, McAuliffe E (2011) Access to health care of persons with disabilities as an indicator of equity in health systems. Open Med 5(1): 10-12.
14. Office of the United Nations High Commissioner for Human Rights (1989) Convention on the Rights of the Child. Available: http://www2.ohchr.org/english/law/crc.htm. Accessed: June 14, 2011.
15. United Nations (2006) Convention on the Rights of Persons with Disabilities. Available: <http://www.un.org/disabilities/convention/conventionfull.shtml>. Accessed: June 14, 2011.
16. Connell BR, Jones M, Mace R, Mueller J, Mullick A, et al. (1997) The Principles of Universal Design. Available: <http://www.ncsu.edu/www/ncsu/design/sod5/cud/about_ud/udprinciplestext.htm>. Accessed: April 29, 2012.
17. Organization of American States (1999) Additional Protocol to the American Convention on Human Rights in the Area of Economic, Social and Cultural Rights. (“Protocol of San Salvador”). Available: <http://www.unhcr.org/refworld/publisher,OAS,,,3ae6b3b90,0.html>. Accessed: March 20, 2012.
18. Rehabilitation Act [29 U.S.C. § 722]. Available: [http://law.onecle.com/usscode/29/722.html](http://law.onecle.com/uscode/29/722.html). Accessed: March 21, 2012.
19. Ensor T, Cooper S (2004) Overcoming barriers to health service access and influencing the demand side through purchasing. Health, Nutrition and Population (HNP) Discussion Paper.
20. Ensor T, Cooper S (2004) Overcoming barriers to health service access: Influencing the demand side. Health Policy & Planning 19(2): 69-79.
21. Goddard M, Smith P (2001) Equity of access to health care services: Theory and evidence from the UK. Social Science & Medicine 53(9): 1149-62.
22. European Union (2000) Charter of Fundamental Rights of the European Union (2000/C 364/01). Available: <http://www.unhcr.org/refworld/docid/3ae6b3b70.html>. Accessed: March 20, 2012.
23. United Nations (1969) Declaration on Social Progress and Development. Available: <http://daccess-dds-ny.un.org/doc/RESOLUTION/GEN/NR0/256/76/IMG/NR025676.pdf?OpenElement>. Accessed: March 20, 2012.
24. Turnbull HR, Beegle G, Stowe MJ (2001) The core concepts of disability policy affecting families who have children with disabilities. Journal of Disability Policy Studies 12(3): 133-43.
25. Sen A (2009) The idea of justice. London: Allen Lane.
26. United Nations (1971) Declaration on the Rights of Mentally Retarded Persons. Available: <http://www2.ohchr.org/english/law/pdf/res2856.pdf>. Accessed: March 21, 2012.
27. Constitution of Venezuela; Art 81 (1999). Available: http://axisoflogic.com/artman/publish/Article_29878.shtml. Accessed: March 21, 2012.
28. World Health Organization (2005) The Bangkok Charter for Health Promotion in a Globalized World. Available: <http://www.who.int/healthpromotion/conferences/6gchp/hpr_050829_%20BCHP.pdf>. Accessed: March 21, 2012.
29. Alma-Ata (1978) Declaration of Alma-Ata. International Conference on Primary Health Care, Alma-Ata, USSR.
30. Gostin L, Mann JM, Gostin L (1994) Towards the development of a human rights impact assessment for the formulation and evaluation of public health policies. Health & Human Rights 1(1): 58-80.
31. Braveman P (2003) Monitoring equity in health and healthcare: A conceptual framework. Journal of Health, Population & Nutrition 21(3): 181-192.
32. Walt G, Shiffman J, Schneider H, Murray SF, Brugha R, et al. (2008) ‘Doing’ health policy analysis: Methodological and conceptual reflections and challenges. Health Policy & Planning 23(5): 308-317.
33. Rifkin SB (2003) A framework linking community empowerment and health equity: It is a matter of CHOICE. Journal of Health, Population & Nutrition 21(3): 168-180.
34. Braveman P, Gruskin S (2003) Poverty, equity, human rights and health. Bull World Health Organ 81(7): 539-45.
35. Thiede M, McIntyre D (2008) Information, communication, and equitable access to health care: A conceptual note. Cadernos de Saúde Pública, Rio de Janeiro 24(5): 1168-73.
36. MacLachlan M, Carr SC, McAuliffe E (2010) The aid triangle: Recognizing the human dynamics of dominance, justice and identity*.* London: Zed.
37. Iannantuono A, Eyles J (1997) Meanings in policy: A textual analysis of Canada’s “achieving health for all” document. Social Science & Medicine 44(11): 1611-21.
38. Whitehead M (2000) The Concepts and Principles of Equity and Health. World Health Organization Regional Office for Europe, Copenhagen.
39. Friel S (2009) Health Equity in Australia: A Policy Framework Based on Action on the Social Determinants of Obesity, Alcohol and Tobacco. The Australian National Preventative Health Taskforce.
40. Marmot M (2007) Achieving health equity: From root causes to fair outcomes. Lancet 370(9593): 1153-63.
41. World Health Organization (2010) Equity, Social Determinants and Public Health Programmes. World Health Organization.
42. World Health Organization (2010) The Case for Change. Background Paper for the Conference: Better Health, Better Lives: Children and Young People with Intellectual Disabilities and their Families. World Health Organization Regional Office for Europe.
43. United Nations (1965) International Convention on the Elimination of All Forms of Racial Discrimination. Available: <http://www.state.gov/documents/organization/100294.pdf>. Accessed: March 20, 2012.
44. United Nations (1995) Declaration on the Occasion of the Fiftieth Anniversary of the United Nations. Available: <http://www.un.org/documents/ga/res/50/a50r006.htm>. Accessed: March 20, 2012.
45. Developmental Disabilities Assistance and Bill of Rights Act of 2000 [42 U.S.C. §§ 15001 et seq.]. Available: <http://uscode.house.gov/download/pls/42C144.txt>. Accessed: March 21, 2012.
46. Makwiza I, Nyirenda L, Bongololo G, Banda T, Chimzizi R, et al. (2009) Who has access to counselling and testing and anti-retroviral therapy in Malawi – an equity analysis. International Journal for Equity in Health 8(13).
47. Bloom G (2001) Equity in health in unequal societies: Meeting health needs in contexts of social change. Health Policy 57: 205-224.
48. Backman G, Hunt P, Khosla R, Jaramillo-Strouss C, Fikre BM, et al. (2008) Health systems and the right to health: An assessment of 194 countries. Lancet 372(9655): 2047-85.
49. Maulik PK, Darmstadt GL (2007) Childhood disability in low- and middle-income countries: Overview of screening, prevention, services, legislation, and epidemiology. Pediatrics 120(1).
50. United Nations (2011) Political Declaration on HIV/AIDS: Intensifying our Efforts to Eliminate HIV/AIDS. Available: <http://www.unaids.org/en/media/unaids/contentassets/documents/document/2011/06/20110610_un_a-res-65-277_en.pdf>. Accessed: March 20, 2012.
51. World Health Organization (2008) International Health Regulations (2005) (2^nd^ ed.). Available: <http://whqlibdoc.who.int/publications/2008/9789241580410_eng.pdf>. Accessed: March 20, 2012.
52. Minister of Health v. Treatment Action Campaign (2002) 5 SA 721 (CC) (South Africa). Available: <http://www.right-to-education.org/node/661>. Accessed: March 21.
53. Council of Europe (1996) European Social Charter. Available: <http://www.coe.int/t/dGHl/monitoring/Socialcharter/Presentation/ESCRBooklet/English.pdf>. Accessed: March 21, 2012.
54. United Nations (1990) International Convention on the Protection of the Rights of All Migrant Workers and Members of their Families. Available: <http://www2.ohchr.org/english/law/pdf/cmw.pdf>. Accessed: March 21, 2012.
55. Pan American Health Organization (2008) Human Rights and Health: Persons with Disabilities. Pan American Health Organization.
56. Hunt P (2007) Report of the Special Rapporteur on the Right of Everyone to the Enjoyment of the Highest Attainable Standard of Physical and Mental Health*.* United Nations General Assembly.
57. World Health Organization (2002) 25 Questions & Answers on Health & Human Rights. Health & Human Rights Publication Series 1.
58. Constitution of Albania; Art 59 (1998). Available: <http://www.president.al/english/pub/doc/Albanian%20Constitution.pdf>. Accessed: March 21, 2012.
59. MacLachlan M (2011) Rehabilitation psychology and global health. In: Kennedy P, editor. Oxford handbook of rehabilitation psychology. Oxford: Oxford University Press.
60. United Nations (2007) United Nations Declaration on the Rights of Indigenous Peoples. Accessed: <http://daccess-dds-ny.un.org/doc/UNDOC/GEN/N06/512/07/PDF/N0651207.pdf?OpenElement>. Available: March 21, 2012.
61. National Assembly of Québec (1975) Charter of Human Rights and Freedoms. Available: <http://www2.publicationsduquebec.gouv.qc.ca/dynamicSearch/telecharge.php?type=2&file=/C_12/C12_A.html>. Accessed: March 21, 2012.
62. United Nations (1948) The Universal Declaration of Human Rights. Available: <http://www.un.org/en/documents/udhr/>. Accessed: March 21, 2012.
63. Goudge J, Gilson L, Russell S, Gumede T, Mills A (2009) Affordability, availability and acceptability barriers to health care for the chronically ill: Longitudinal case studies from South Africa. BMC Health Services Research 9(75).
64. World Health Organization, Regional Office for the Western Pacific (2007) Reaching the Poor: Challenges for Child Health in the Western Pacific Region. World Health Organization.
65. Perry HB, King-Schultz LW, Aftab AS, Bryant JH (2007) Health equity issues at the local level: Socio-geography, access, and health outcomes in the service area of the Hôpital Albert Schweitzer-Haiti. International Journal for Equity in Health 6(7).
66. Shaw CD, Kalo I (2002) A Background for National Quality Policies in Health Systems. World Health Organization.
67. Gruskin S, Tarantola D (2008) Health and human rights: Overview. International Encyclopaedia of Public Health 3: 137-146.
68. World Health Organization, Regional Office for South-East Asia (2010) Convention on the Rights of Persons with Disabilities: Roles and Responsibilities of the Health Sector: Information for Policy-Makers. World Health Organization.
69. MacLachlan M, Mji G, McLaren P, Gcaza S (2009, Volume 1) Realising the Rights of Persons with Disabilities in Africa. Special Issue of Disability & Rehabilitation 1.
70. United Nations (2008) Political Declaration on Africa’s Development Needs. Available: <http://www.unhcr.org/refworld/topic,4565c22529,458aa6c22,4912b7fa2,0,,,.html>. Accessed: March 21, 2012.
71. La Rosa-Salas V, Tricas-Sauras S (2008) Equity in health care. Cuadernos de Bioetica 19(66): 355-68.
72. Mann JM, Gostin L, Gruskin S, Brennan T, Lazzarini Z, et al. (1994) Health and human rights. Health & Human Rights 1(1): 6-23.
73. Zere E, Mandlhate C, Mbeeli T, Shangula K, Mutirua K, et al. (2007) Equity in health care in Namibia: Developing a needs-based resource allocation formula using principal components analysis. International Journal for Equity in Health 6(3).
74. Stewart Williams JA, Byles JE, Inder KJ (2010) Equity of access to cardiac rehabilitation: The role of system factors. International Journal for Equity in Health 9(2).
75. Ong KS, Kelaher M, Anderson I, Carter R (2009) A cost-based equity weight for use in the economic evaluation of primary health care interventions: Case study of the Australian Indigenous population. International Journal for Equity in Health 8(34).
76. Penchansky R, Thomas JW (1981) The concept of access: Definition and relationship to consumer satisfaction. Med Care 19(2): 127-40.
77. Turmen T, Troedsson H, Stahlhofer M (2001) A human rights approach to public health: WHO capacity building in the area of children’s rights. Health & Human Rights 5(2): 147-154.
78. World Health Organization (2010) A Conceptual Framework for Action on the Social Determinants of Health. Social Determinants of Health Discussion Paper 2: Debates, Policy & Practice, Case Studies*.* World Health Organization*.* Available: <http://www.ossyr.org.ar/pdf/bibliografia/131.pdf>. Accessed: May 05, 2011.
79. Tamburlini G (2004) Promoting equity in health. Health Policy & Development 2(3): 186-191.
80. McIntyre D, Gilson L (2002) Putting equity in health back onto the social policy agenda: experience from South Africa. Social Science & Medicine 54(11): 1637-56.
81. Odaga J (2004) From Alma Ata to Millennium Development Goals: To what extent has equity been achieved? Health Policy & Development 2(1): 1-6.
82. United Nations (2010) Keeping the promise: united to achieve the Millennium Development Goals [Resolution adopted by the General Assembly 2010]. Available: <http://daccess-dds-ny.un.org/doc/UNDOC/GEN/N10/512/60/PDF/N1051260.pdf?OpenElement>. Accessed: March 21, 2012.
83. United Nations (2001) Durban Declaration and Programme of Action. Available: <http://www.un.org/en/ga/durbanmeeting2011/pdf/DDPA_full_text.pdf>. Accessed: March 21, 2012.
84. Constitution of Republic of Ecuador; Art 66 (2008). Available: <http://pdba.georgetown.edu/Constitutions/Ecuador/english08.html>. Accessed: March 21, 2012.
85. Constitution of Venezuela; Art 84 (1999). Available: <http://axisoflogic.com/artman/publish/Article_29878.shtml>. Accessed: March 21, 2012.
86. Jacobs R, Smith PC, Street A (2006) Measuring efficiency in health care: Analytical techniques and health policy*.* New York: Cambridge University Press.
87. Roberts MJ, Reich MR (2002) Ethical analysis in public health. Lancet 359(9311): 1055-59.
88. Constitution of Colombia; Art 49 (1991). Available: <http://confinder.richmond.edu/admin/docs/colombia_const2.pdf>. Accessed: March 21, 2012.
89. Constitution of Peru; Art 11 (1993). Available: <http://www.congreso.gob.pe/_ingles/CONSTITUTION_29_08_08.pdf>. Accessed: March 21, 2012.
90. Constitution of the Portuguese Republic; Art 64 (2005). Available: <http://app.parlamento.pt/site_antigo/ingles/cons_leg/Constitution_VII_revisao_definitive.pdf>. Accessed: March 21, 2012.
