# Supplementary material for: Inclusion and Human Rights in Health Policies: Comparative and Benchmarking Analysis of 51 Policies from Malawi, Sudan, South Africa and Namibia
Source: PLoS One. 2012 May 23;7(5):e35864. doi: 10.1371/journal.pone.0035864 (PMC3359320; doi:10.1371/journal.pone.0035864)
Supplement: Table S2 — EquiFrame Vulnerable Group Definitions. (DOCX) [file pone.0035864.s002.docx]

**Table S2: *EquiFrame* Vulnerable Group Definitions**

| **Supporting Literature** |  |  | **Attributes or Definitions** | **Vulnerable Group** | **No.** |
| --- | --- | --- | --- | --- | --- |
| 1, 2, 3, 4, 5, 6, 7, 8, 9, 10, 11, 12, 13, 14, 15, 16, 17, 18, 19 |  |  | Referring to poor people or people living in poverty | **Limited Resources** | 1. |
| 12, 20, 21, 22, 23 |  |  | Referring to people with one of the top 10 illnesses, identified by WHO, as occurring within the relevant country | **Increased Relative Risk For Morbidity** | 2. |
| 3, 5, 6, 8, 10, 11, 13, 15, 20, 21, 22, 24, 25, 26, 27 |  |  | Referring to factors affecting maternal and child health (0-5 years) | **Mother Child Mortality** | 3. |
| 1, 2, 3, 4, 5, 6, 12, 17, 20, 28, 29, 30, 31, 32 |  |  | Referring to households headed by a woman | **Women Headed Household** | 4. |
| 24, 33, 34, 35 |  |  | Referring to children marginalized by special contexts, such as orphans or street children | **Children (with special needs)** | 5. |
| 3, 28, 30, 36, 37 |  |  | Referring to older age | **Aged** | 6. |
| 3, 10, 20, 28, 33, 38 |  |  | Referring to younger age without identifying gender | **Youth** | 7. |
| 2, 3, 6, 10, 12, 20, 39, 40, 41 |  |  | Referring to non-majority groups in terms of culture, race or ethnic identity | **Ethnic Minorities** | 8. |
| 20, 40 |  |  | Referring to people who, because of civil unrest or unsustainable livelihoods, have been displaced from their previous residence | **Displaced Populations** | 9. |
| 1, 4, 6, 36, 42, 43 |  |  | Referring to people living far from health services, either in time or distance | **Living Away from Services** | 10. |
| 6, 9 |  |  | Referring to people who have an illness which requires continuing need for care | **Suffering from Chronic Illness** | 11. |
| 3, 13, 20, 28, 34, 44, 45, 46, 47, 48 |  |  | Referring to persons with disabilities, including physical, sensory, intellectual or mental health conditions, and including synonyms of disability | **Disabled** | 12. |

**References**

1. Ensor T, Cooper S (2004) Overcoming barriers to health service access and influencing the demand side through purchasing. Health, Nutrition and Population (HNP) Discussion Paper.
2. Braveman P (2003) Monitoring equity in health and healthcare: A conceptual framework. Journal of Health, Population & Nutrition 21(3): 181-192.
3. United Nations Economic and Social Council (2000) Substantive Issues Arising in the Implementation of the International Covenant on Economic, Social and Cultural Rights. General Comment No. 14. The Right to the Highest Attainable Standard of Health (Article 12 of the International Covenant on Economic, Social and Cultural Rights). Available: <http://www.unhchr.ch/tbs/doc.nsf/%28symbol%29/E.C.12.2000.4.En>. Accessed: April 29, 2012.
4. Ensor T, Cooper S (2004) Overcoming barriers to health service access: Influencing the demand side. Health Policy & Planning 19(2): 69-79.
5. Marmot M (2007) Achieving health equity: From root causes to fair outcomes. Lancet 370(9593): 1153-63.
6. World Health Organization (2010) Equity, Social Determinants and Public Health Programmes. World Health Organization.
7. Bloom G (2001) Equity in health in unequal societies: Meeting health needs in contexts of social change. Health Policy 57: 205-224.
8. Hunt P (2007) Report of the Special Rapporteur on the Right of Everyone to the Enjoyment of the Highest Attainable Standard of Physical and Mental Health*.* United Nations General Assembly.
9. Goudge J, Gilson L, Russell S, Gumede T, Mills A (2009) Affordability, availability and acceptability barriers to health care for the chronically ill: Longitudinal case studies from South Africa. BMC Health Services Research 9(75).
10. World Health Organization, Regional Office for the Western Pacific (2007) Reaching the Poor: Challenges for Child Health in the Western Pacific Region. World Health Organization.
11. Zere E, Mandlhate C, Mbeeli T, Shangula K, Mutirua K, et al. (2007) Equity in health care in Namibia: Developing a needs-based resource allocation formula using principal components analysis. International Journal for Equity in Health 6(3).
12. World Health Organization (2010) A Conceptual Framework for Action on the Social Determinants of Health. Social Determinants of Health Discussion Paper 2: Debates, Policy & Practice, Case Studies*.* World Health Organization*.* Available: <http://www.ossyr.org.ar/pdf/bibliografia/131.pdf>. Accessed: May 05, 2011.
13. Tamburlini G (2004) Promoting equity in health. Health Policy & Development 2(3): 186-191.
14. McIntyre D, Gilson L (2002) Putting equity in health back onto the social policy agenda: experience from South Africa. Social Science & Medicine 54(11): 1637-56.
15. Lanre-Abass BA (2008) Poverty and maternal mortality in Nigeria: Towards a more viable ethics of modern medical practice. International Journal for Equity in Health 7(11).
16. Ridde V (2008) “The problem of the worst-off is dealt with after all other issues”: The equity and health policy implementation gap in Burkina Faso. Social Science & Medicine 66(6): 1368-78.
17. Gilson L, Kalyalya D, Kuchler F, Lake S, Oranga H, et al. (2001) Strategies for promoting equity: Experience with community financing in three African countries. Health Policy 58(1): 37-67.
18. World Health Organization, World Bank Working Group on Child Health and Poverty (2001) Better Health for Poor Children: A Special Report. World Health Organization/ World Bank.
19. Roy K, Hill Howard D (2007) Equity in out-of-pocket payments for hospital care: Evidence from India. Health Policy 80(2): 297-307.
20. Office of the United Nations High Commissioner for Human Rights, World Health Organization (2008) The Right to Health. United Nations, Geneva.
21. Braveman P, Gruskin S (2003) Poverty, equity, human rights and health. Bull World Health Organ 81(7): 539-45.
22. Mugisha J (2004) Reduction of user fees in the private-not-for-profit hospitals in Uganda: Implications for equity and sustainability. Health Policy & Development 2(3): 209-216.
23. MacNaughton G (2004) Health and Human Rights Working Paper Series No 5: Women’s Human Rights related to Health-Care Services in the Context of HIV/AIDS. The International Centre for the Legal Protection of Human Rights, London.
24. Maulik PK, Darmstadt GL (2007) Childhood disability in low- and middle-income countries: Overview of screening, prevention, services, legislation, and epidemiology. Pediatrics 120(1).
25. World Health Organization (2005) Child Health and Development: Child Survival in the South-East Asia Region. Available: <http://www.searo.who.int/en/Section13/Section37/Section135.htm>. Accessed: December 01, 2010.
26. EQUINET (2009) Taking Forward the Equity Watch in East and Southern Africa: Regional Methods Workshop Report*.* Available: <http://www.equinetafrica.org/bibl/docs/EWmtg%20repNov09.pdf>. Accessed: May 04, 2011.
27. Tugwell P, Petticrew M, Robinson V, Kristjansson E, Maxwell L, et al. (2006) Cochrane and Campbell Collaborations, and health equity. Lancet 367(9517): 1128-1130.
28. Hunt P, Backman G (2008) Health systems and the right to the highest attainable standard of health*.* Health & Human Rights 10(1): 81-92.
29. Rifkin SB (2003) A framework linking community empowerment and health equity: It is a matter of CHOICE. Journal of Health, Population & Nutrition 21(3): 168-180.
30. Whitehead M (2000) The Concepts and Principles of Equity and Health. World Health Organization Regional Office for Europe, Copenhagen.
31. Loeb M, Grut L (2005) Women with Disabilities Sharing Knowledge: Education, Employment, Reproductive History. SINTEF Report.
32. World Health Organization (2002) Integrating Gender Perspectives in the Work of WHO: WHO Gender Policy. World Health Organization.
33. World Health Organization (2010) The Case for Change. Background Paper for the Conference: Better Health, Better Lives: Children and Young People with Intellectual Disabilities and their Families. World Health Organization Regional Office for Europe.
34. Pan American Health Organization (2008) Human Rights and Health: Persons with Disabilities. Pan American Health Organization.
35. Panter-Brick C (2002) Street children, human rights, and public health: A critique and future directions. Annual Review of Anthropology 31: 147-71.
36. La Rosa-Salas V, Tricas-Sauras S (2008) Equity in health care. Cuadernos de Bioetica 19(66): 355-68.
37. Braveman P (2006) Health disparities and health equity: Concepts and measurement. Annu Rev Public Health 27: 167-94.
38. Doebbler CF (2001) The right to health of children and the World Bank. Health & Human Rights 5(2): 120-146.
39. MacLachlan M (2006) Culture & health: A critical perspective towards global health (Second Edition). Chichester: Wiley.
40. World Health Organization (2002) 25 Questions & Answers on Health & Human Rights. Health & Human Rights Publication Series 1.
41. Dixon Woods M, Kirk D, Agarwal S, Annandale E, Arthur T, et al. (2005) Vulnerable Groups and Access to Healthcare: A Critical Interpretive Review. National Co-ordinating Centre for NHS Service Delivery and Organization R & D.
42. Makwiza I, Nyirenda L, Bongololo G, Banda T, Chimzizi R, et al. (2009) Who has access to counselling and testing and anti-retroviral therapy in Malawi – an equity analysis. International Journal for Equity in Health 8(13).
43. Odaga J (2004) From Alma Ata to Millennium Development Goals: To what extent has equity been achieved? Health Policy & Development 2(1): 1-6.
44. Wiman R, Helander E, Westland J (2002) Meeting the needs of people with disabilities – New approaches in the health sector. World Bank. Available: http://bvs.per.paho.org/texcom/cd048370/meeting.pdf. Accessed: May 04, 2011.
45. MacLachlan M, Mji G, McLaren P, Gcaza S (2009, Volume 1) Realising the Rights of Persons with Disabilities in Africa. Special Issue of Disability & Rehabilitation 1.
46. MacLachlan M, Swartz L (2009) Disability & international development: Towards inclusive global health. New York: Springer.
47. ILO (2002) Disability and Poverty Reduction Strategies: How to Ensure that Access of Persons with Disabilities to Decent and Productive Work is Part of the PRSP Process. ILO.
48. Marks D (2001) Disability: Controversial debates and psychosocial perspectives. UK: Routledge.
